# Associations between children’s diagnosed mental disorders and educational achievements in Sweden

**DOI:** 10.1177/14034948221089056

**Published:** 2022-04-13

**Authors:** Cristian Bortes, Karina Nilsson, Mattias Strandh

**Affiliations:** 1 Department of Social Work, Umeå University, Sweden; 2 Department of Sociology, Umeå University, Sweden

**Keywords:** Mental disorders, educational achievement, register data, Sweden, sex differences

## Abstract

**Aims:**

To examine associations between multiple clinically diagnosed mental disorders among children in Sweden and educational achievements at the end of ninth grade.

**Methods:**

Data from Swedish administrative registers were utilised. Diagnoses of specific mental disorders (unipolar depression, mood, anxiety, obsessive compulsive, eating, attention deficit hyperactivity disorder) were used as exposure variables. Educational achievements were assessed in terms of teacher-assigned school grades and eligibility for upper secondary education. The sample comprised 266,664 individuals (49% females) born in 2000 to 2002 who were alive and resident in Sweden in 2017. Exposed and unexposed individuals were compared in terms of outcome variables by fitting linear and logistic regression models.

**Results:**

The results revealed negative associations between all the examined mental disorders and educational achievements, except for positive associations between eating disorders and grades among female students. Attention deficit hyperactivity disorder was the most strongly associated disorder in terms of non-successful completion of compulsory education, among both male and female students (odds ratio (OR): 3.58 (95% confidence interval (CI), 3.42 to 3.74) and 4.31 (95% CI, 4.07 to 4.57), respectively). This was followed by unipolar depression among males (OR: 2.92 (95% CI, 2.60 to 3.28)) and anxiety disorder among females (OR: 2.68 (95% CI, 2.49 to 2.88)). Obsessive compulsive disorder had the weakest negative association with educational achievements among both males (OR: 1.48 (95% CI, 1.01 to 2.17)) and females (OR: 1.38 (95% CI, 1.11 to 1.72)).

**Conclusions:**

**Specific diagnosed mental disorders have varying, largely disadvantageous, associations with educational achievements of students in Sweden that differ between males and females.**

## Introduction

Children’s education and their mental health are highly important for societies’ welfare and public health [1,2]. For individuals, higher educational attainment is positively associated with various desirable outcomes in adulthood, for example, a steady foothold on the labour market and capabilities of both self-governance and social participation [3]. Education is also a fundamental determinant of future health outcomes [4,5]. However, in recent decades there have been increases in frequencies of mental health problems among children and youths [6], including clinical diagnosis and treatment of psychiatric disorders [7]. These trends are worrying as research has shown clear negative effects of mental illness among children on their learning in school [8–11]. However, empirical studies on associations between clinically diagnosed psychiatric mental disorders and objectively measured educational outcomes in Sweden are rare.

The most common mental disorders among children and youths are anxiety disorders, followed by behavioural and mood disorders [12]. Anxiety disorders, including, for example, social phobia, have negative effects on psychosocial functioning, which may lead to withdrawal from school [13]. Depression, characterised by negative self-perceptions, low energy and difficulties in concentrating [14], may also affect ability to perform in school. A recent systematic review and meta-analysis detected a weak negative association between depression of children or adolescents and lower educational achievement [8]. However, in most of the studies included in the review symptoms of depression were used as the exposure, rather than diagnoses. Moreover, of all the 31 studies included, only one was conducted in Sweden [15], which used graduation from higher education as the outcome. While an important contribution, the study was limited by methodological issues, such as small sample size and potential selection bias. Hence, the significance of clinically diagnosed depression for educational achievement in the Swedish context remains unclear.

Another mental disorder that has increased in administrative (i.e. clinically diagnosed or recorded) prevalence among children in recent years is attention deficit hyperactivity disorder (ADHD) [16]. ADHD is a neurodevelopmental disorder characterised by difficulties in sustaining attention, restless overactivity and impulse control [17]. These symptoms tend to impair school functioning and the required abilities for learning and studying in a traditional teaching environment. There is substantial evidence of a negative association between ADHD symptoms and learning outcomes, both in Sweden and elsewhere [8,10,18].

To date, most studies on the topic have focused on single disorders, although comorbidity of mental disorders, that is, co-existence of two or more psychiatric disorders, is very common [19]. It is therefore essential to consider the differential impact of several mental disorders on educational achievement, taking comorbidity into account.

A recent study from Denmark compared educational achievements in the final examination of compulsory schooling between individuals with and without the full recognised spectrum of mental disorders [20]. The results showed that almost all of the 29 specific mental disorders considered were negatively associated with likelihood of taking the examination at the end of ninth grade. There were also associations between specific disorders and lower than average mean grades for this examination, and some disorders affected the performance of female students more than that of male students.

The aim of the present study was to extend these findings by examining associations between the most common clinically diagnosed mental disorders among children in Sweden and their educational achievements at the end of ninth grade (at the age of 15–16 years). Differences between countries in healthcare, education and related support systems (e.g. the organisation of school health services) limit the transferability and comparability of results. Hence, the association between mental disorders and educational outcomes that are relevant in the Swedish education system is an issue that must be empirically resolved in its own right, rather than inferred from results of studies elsewhere. Thus, the present study adds to existing literature by documenting these associations in the Swedish context. Furthermore, while Dalsgaard et al. [20] examined the ninth grade final examination in two subjects (Danish and mathematics) as outcome, the current study examined outcomes that are core indicators of educational achievement in the Swedish education system; the overall grade sum that students obtain in ninth grade serves as a summary measure of school-relevant skills and an aggregated measure of students’ assessments in 17 subjects by multiple teachers. Additionally, we also examined eligibility for an upper secondary education as an outcome (see below for details).

In addition to depression and ADHD, discussed above, we also considered obsessive compulsive disorder (OCD) and mood, anxiety and eating disorders. These were the six most common diagnoses that individuals in our study population (see below) received when in contact with psychiatric outpatient care units. By considering multiple mental disorders simultaneously, the study provides evidence on those that most strongly affect children’s educational achievements and thus may assist school staff, healthcare professionals and others concerned with their health, wellbeing and outcomes.

## Methods

### Data sources and study population

Data for this study were obtained from the Umeå SIMSAM Lab [21], which compiles prospectively collected individual-level data from several Swedish administrative registers covering the entire population. One source used was the Total Population Register, which is used for the production of statistics on the size and composition of the population and forms the basis for statistics on, for example, domestic and foreign relocations, births, deaths, marriages and divorces. The other sources of information were the National Patient Register (NPR), which contains information on all inpatient hospital care in Sweden [22]. The register also covers outpatient doctor visits including psychiatric care provided by both private and public caregivers since 2001. We used information on all contacts with outpatient psychiatric care units. Data regarding each contact include diagnoses of mental disorders according to the 10th revision of the International Classification of Diseases (ICD-10). We also used information on school grades from the Swedish National Pupil Register and socioeconomic information from the Longitudinal Integrated Database for Health Insurance and Labour Market Studies [23].

The study population comprised all individuals born in 2000, 2001 and 2002 who were alive and resident in Sweden in 2017 (*N* = 348,230). These cohorts were selected to analyse the latest available data. In the analyses, we excluded individuals for whom school grades at the end of compulsory education (due to lack of completion) and/or data on socioeconomic status were missing (81,566 individuals were lost). The final analytical sample consisted of 266,664 individuals. The Regional Ethical Vetting Board in Umeå approved all research based on data from the Umeå SIMSAM Lab, including the study presented here (Dnr. 2018-99-31).

### Exposure variables

The exposures were defined as receipt of diagnosis of the following six mental disorders according to the ICD-10, as recorded in the NPR: mood disorder (ICD-10 codes F30–F39: includes all mood disorders except unipolar depression); unipolar depression (ICD-10 codes F32–F33); anxiety disorder (ICD-10 codes F40–F48), including obsessive compulsive disorder (OCD) (ICD-10 code F42); eating disorder (ICD-10 code F50); and ADHD (ICD-10 code F90). All diagnoses were defined as at least one registered contact(s)/diagnosis in the NPR any time before an individual received their final grade in ninth grade. The unexposed group had none of the studied or other mental disorders not included in the study, for example, schizophrenia.

### Outcome variables

Two variables were used to measure educational achievement: overall grade sum and eligibility for upper secondary education. Both are based on the school grades assigned to students by teachers in accordance with the Swedish grading system. The first indicates students’ levels of educational performance, while the second reveals problems with completing compulsory education. Compulsory education in Sweden lasts until age 16 years on average. On completion of compulsory schooling, students are assigned a final school grade obtained by summing their 17 best subject grades in the final year. For each subject, a student can obtain a grade ranging from 0, indicating that the minimal knowledge requirements for that subject were not achieved, to 20, so the possible sum ranges from 0 to 340. We standardised the grades into *z*-scores (mean = 0, standard deviation = 1). Thus, estimated standardised mean grade differences between exposed and unexposed individuals were measured in terms of standard deviations. Regarding the second outcome, admission requirements for upper secondary education differ between vocational and academic programmes. Eligibility for a vocational programme requires a pass grade in three core subjects (Swedish, English and mathematics) and at least five other subjects (thus eight subjects in total). Eligibility for an academic programme requires pass grades in Swedish, English, mathematics and at least nine other subjects (thus 12 subjects in total). Students were dichotomised, irrespective of their choice of programme orientation, as either eligible or ineligible for access to upper secondary education.

### Statistical analyses

Exposed and unexposed individuals were compared in terms of the outcome variables by fitting linear and logistic regression models. We also investigated effect modification by sex using stratified models. Socioeconomic position is known to be strongly associated with both mental health and educational achievement. Thus, models were also adjusted for socioeconomic position, measured by family income, and maternal and paternal educational level, classified as compulsory school (nine years or less), secondary school (10–12 years) and university (> 12 years). First, we estimated crude models of associations between each diagnosis and each outcome, which were then adjusted for socioeconomic status (these models are presented in the Supplemental material online). To account for comorbidity, we then included all diagnoses in a single model for each outcome to detect potential effects of comorbidity. That is, we adjusted the model for all diagnoses. In this way, the analysis takes into account all diagnoses a child has potentially received during the study period and provides adjusted estimates. Multicollinearity tests yielded a variance inflation factor below 2 for each variable, which is below critical levels and indicates that there are no problems with multicollinearity. Results of the latter, fully adjusted, models are presented below in the form of differences in standardised mean grades and odds ratios (ORs) of ineligibility for upper secondary education, in both cases with 95% confidence intervals. Stata v. 14 was used for all analyses.

## Results

Of the total study sample, 15.6% of males and 17.4% of females had been in contact with an outpatient psychiatric unit and received a clinical diagnosis of at least one of the mental disorders considered in this study. The most common mental disorders were ADHD followed by anxiety disorder and unipolar depression (Table I). Further descriptive statistics are provided in the Supplemental material (eTable Ia and eTable Ib), showing how many individuals have more than one diagnosis and which diagnoses are occurring together.

**Table I.** Standardised mean grade differences between individuals with and without mental disorders.

Diagnosis	Male (*n* = 136,539)	Female (*n* = 130,125)	Standardised mean grade difference (95% CI)^a^		*p* value for difference by sex^b^
			Male	Female	
Mood disorder	158	320	−0.35 (−0.47 to −0.22)	−0.42 (−0.50 to −0.33)	0.23
Unipolar depression	1851	4493	−0.62 (−0.66 to −0.58)	−0.46 (−0.48 to −0.44)	<0.001
Anxiety disorder	2667	6405	−0.47 (−0.50 to −0.44)	−0.53 (−0.55 to −0.51)	0.003
OCD	647	749	−0.03 (−0.09 to 0.03)	−0.08 (−0.13 to −0.02)	0.046
Eating disorder	234	2376	0.01 (−0.09 to 0.11)	0.19 (0.16 to 0.23)	0.001
ADHD	15,699	8280	−0.58 (−0.59 to −0.57)	−0.74 (−0.76 to −0.72)	<0.001

a Model adjusted for all the diagnoses, family disposable income and parental educational level.

b *p* value of the interaction term indicating statistical significance of the difference.

CI: confidence interval; OCD: obsessive compulsive disorder; ADHD: attention deficit hyperactivity disorder

### Standardised mean grade differences

Table I shows the standardised mean grade differences (expressed as standard deviations) between individuals with and without diagnosed mental disorders. Unipolar depression was the mental disorder associated with the lowest mean grade for male students. The disorders associated with the second and third lowest mean grades for male students were ADHD and anxiety disorder. ADHD was the mental disorder associated with the lowest mean grades for female students. The mental disorders associated with the second and third lowest mean grades for females were anxiety disorder and unipolar depression. Female students with eating disorders had higher grades, on average, than the general population. Effect modification by sex was found for all mental disorders, except mood disorder, as the statistical effect of mental disorders on school grades was larger among females. The associations displayed in Table I were weaker than those in crude models and those adjusted solely for socioeconomic position (cf. Supplemental eTable II).

### Ineligibility for upper secondary education

Exploration of associations between mental disorders and ineligibility for upper secondary education revealed a similar pattern to their associations with standardised mean grades. Table II shows that individuals with mental disorders were less likely to be eligible for upper secondary education than the general population, but strengths of the associations between specific disorders and ineligibility varied substantially. Male and female students with ADHD had the highest ORs for ineligibility compared with their counterparts with no diagnosed mental disorders. The disorders associated with the second and third highest ORs for ineligibility among male students were unipolar depression and anxiety disorder. The disorders associated with the second and third highest ORs for ineligibility among female students were anxiety disorder and unipolar depression. In contrast to the standardised mean grade difference, males with eating disorder had a higher OR of ineligibility for upper secondary education. Females with eating disorder had a lower OR for ineligibility, although not statistically significant. Effect modification by sex was found for ADHD, which increased the likelihood of ineligibility significantly more for females than males, while unipolar depression and eating disorder increased the likelihood significantly more for males. The ORs displayed in Table II were much lower than those obtained from the crude models and models adjusted solely for socioeconomic position (cf. Supplemental eTable III).

**Table II.** Associations between mental disorders and ineligibility for upper secondary education.

	ORs of ineligibility (95% CI)^a^		*p* value for difference by sex^b^
	Male	Female	
Mood disorder	1.89 (1.30 to 2.74)	2.05 (1.55 to 2.69)	0.92
Unipolar depression	2.92 (2.60 to 3.28)	2.11 (1.93 to 2.30)	<0.001
Anxiety disorder	2.62 (2.37 to 2.89)	2.68 (2.49 to 2.88)	0.75
OCD	1.29 (1.03 to 1.62)	1.38 (1.11 to 1.72)	0.64
Eating disorder	1.48 (1.01 to 2.17)	0.86 (0.73 to 1.02)	0.006
ADHD	3.58 (3.42 to 3.74)	4.31 (4.07 to 4.57)	<0.001

a Model adjusted for all the diagnoses, family disposable income and parental educational level.

b *p* value of the interaction term indicating statistical significance of the difference.

OR: odds ratio; CI: confidence interval; OCD: obsessive compulsive disorder; ADHD: attention deficit hyperactivity disorder

## Discussion

Results of our investigation showed negative associations between all the mental disorders diagnosed in childhood or adolescence and educational achievements at the end of ninth grade except for eating disorders among females. The results also reveal clear differences in associations between specific diagnoses and the considered outcomes. This clearly shows variations in vulnerability associated with different mental disorders. Taking potential comorbidity into account (in the fully adjusted models) revealed that ADHD was the disorder that was, by far, the most strongly associated with students not successfully completing compulsory education (the outcome ineligibility for upper secondary education), for both male and female students. This was followed by unipolar depression for males and anxiety disorder for females, while OCD had the least negative association with educational achievements (see Table II; cf. Supplemental material). Success in school requires abilities such as cognitive, organisational and time-management skills, as well social skills (for participation in group projects, for example). Symptoms of disorders such as ADHD (inattention, hyperactivity, impulsiveness) and depression (reductions in energy, difficulties in concentrating) clearly impair these skills. By documenting this in Sweden the study adds to the existing body of evidence that mental disorders have a negative overall association with educational achievement, despite substantial variation in support and educational systems across countries [8,9,11].

However, the educational consequences of mental health problems should not be considered in terms of individual impairment alone. Students with mental health problems may experience social stigma, peer discrimination and a lack of understanding from teachers [24]. Studies have shown that teachers prefer students who approach schoolwork with a positive attitude, are organised and not disruptive in the classroom, and they take this into account when evaluating students’ performance [25,26]. Thus, students may be assessed on the basis of more than just their cognitive abilities, including non-cognitive, behavioural traits. Traits such as aggressiveness and disruptiveness (commonly associated with ADHD) or passivity and withdrawal (commonly associated with depression, mood and anxiety disorders) could be interpreted as a lack of motivation and interest in achievement [27,28]. Hence, students’ externalising and internalising symptoms could affect the student–teacher relationship and their evaluation [28], so social responses induced by mental health problems in school settings should also probably be considered when addressing their educational consequences.

The results also showed notable differences in the associations by sex, especially for the outcome overall grade sum. Unipolar depression was more strongly associated with lower than average standardised mean grades among male students, who also were less likely to obtain eligibility for upper secondary education than their female peers with the same disorder. This is consistent with previous findings that depression reduced the likelihood of subsequent graduation from higher education for males, but not females, in Sweden [15]. The negative associations between anxiety disorder, OCD, ADHD and school grades were stronger for female individuals than for their male counterparts with the same diagnoses. As female students generally outperform male students in education [29], this is a highly noteworthy finding. A possible explanation for these sex differences may lie in how girls and boys with mental health problems are perceived by adults in terms of impairment. One study of children with mental health problems found that girls tend to be perceived as less impaired than boys by teachers and parents [30]. Thus, the support given to males and females with mental health problems may differ. Systematic gender-based differences in educational disadvantage associated with diagnosis of particular mental disorders warrant rigorous attention, including potential extents of link with differential social responses from health and school personnel.

Of further note, females who had been diagnosed with eating disorders had higher mean grades than the general population. This is consistent with previous findings that high educational achievement is common in adolescents with eating disorders [20,31,32]. However, mean grades of males diagnosed with eating disorders did not differ from those of the general population, although they were less likely to be eligible for upper secondary education. Thus, there was also clear difference between the sexes in educational outcomes associated with this disorder. The explanation attributed to the positive association found among females is related to the high level of self-oriented perfectionism in the disorder (i.e. inner strivings for perfection), although studies on long-term educational and occupational outcomes show mixed results. A Finnish population cohort study [33] found that women with a history of anorexia nervosa had similar outcomes to unaffected women in terms of education and employment. In contrast, an international multi-site study found that a lower percentage of women with eating disorder in their medical history had a graduate degree or professional training than sex- and age-matched controls [34]. Duration of illness and age of onset are highly likely to play a part in these associations. If the disorder persists for a long time, it may very well be detrimental to subsequent outcomes.

OCD presents in a symptomatically heterogeneous manner and persons with the disorder may exhibit a broad range of functional impairments [35]. Those related to education include dressing and grooming rituals that may result in late arrival at school and difficulties at bedtime. In addition, counting, checking, repeating rituals and intrusive thoughts can impair focus and concentration, and interfere with reading, listening and conversation, thereby impairing school functioning. We found that OCD was the diagnosis that was most weakly associated with the studied outcomes, and previous studies have yielded mixed results. One based on Swedish registry data found that the disorder was associated with decreased achievement across all educational levels [36], while another based on Danish registry data found that it was associated with higher grades [20].

Furthermore, the associations between all the exposures and outcomes were greatly attenuated in the fully adjusted model, that is, when all the diagnoses were included in the same model (cf. Supplemental material). This suggests that comorbidity explains much of the ‘effect’ of individual diagnoses, implying that individuals with psychiatric problems tend to have more than just one problem, which is also confirmed by previous findings that comorbidity in mental disorders is very common [19].

While this study has several strengths, including the longitudinal population-based design and use of national registers with high completeness and validity, it also has some limitations that should be noted. We examined associations of registered diagnoses rather than mental health problems per se. We did not assess the severity or duration of the mental disorders, or the timing of the exposure, that is, the age of onset, which may be important factors for the studied outcomes [36]. Also, poor academic performance may lead to mental disorder diagnosis either by directly triggering symptoms (e.g. anxiety about not doing well in school) or by causing parents to seek evaluation for their child to determine why they are struggling in school. The association between mental health and academic performance could in this sense be regarded as bidirectional. While the directionality of associations was not a focus of the current study, it would have been beneficial to have included information on academic achievement prior to mental disorder diagnosis. Moreover, we adjusted for family income and parental education level as potential confounders. However, there are many other potential confounders. Future studies could consider, for example, age at first diagnosis, disease severity, parental mental or physical disorder, to get more knowledge of the mechanisms behind the associations. Furthermore, the unexposed group were individuals with none of the six mental disorders studied, also those with other mental disorders not included in the study. This is a possible limitation but given that the other mental disorders are relatively unusual they should not affect the results because they make up such a small part of the unexposed population (comparison group). Lastly, records of diagnosis imply subsequent treatment and, in many cases, special support in school [37]. Thus, the reported associations may be underestimates as the performance of some undiagnosed individuals with mental health problems may have been more severely impaired.

Regardless of the strength of such possible underestimation, it is clearly important for both practitioners and future researchers to identify optimal ways to support students to alleviate the negative impact of mental disorders on their schooling. In Sweden, a number of measures have been introduced to help children and adolescents with mental health problems [37]. The results of the current study demonstrate which diagnosis is associated with worst educational outcomes. This informs about the type of mental disorder in need of most support, which in turn could be used in preventing educational disadvantage associated with mental disorders among school-age children. However, it is educational assessments that should form the basis for additional adjustments or special support in school rather than a medical diagnosis. Thus, special support and preventive measures should precede a psychiatric diagnosis. The schools are central actors in this regard and the student health services have particular responsibility for students’ medical, psychological, psychosocial and special educational health. However, the student health services in Sweden have a number of shortcomings. Access to services is limited in most Swedish schools, as the working hours of doctors, nurses and counsellors do not cover the students’ needs [38]. There is also a lack of clear guidelines for student health staff regarding obligations to help students who have mental illness or psychosocial risk environments [38]. There is also poor cooperation between the student health staff and pedagogical staff in many schools, despite its importance [38]. Given the increases in frequencies of mental health problems among school-age children and their implications for the children’s learning there are, despite good intentions, insufficient measures to meet the challenges.

## Conclusion

The study clearly reveals the differential impact of clinically diagnosed mental disorders on educational achievements between male and female students in Sweden, and the varying educational disadvantage associated with specific mental disorders.

## Supplemental Material

sj-docx-1-sjp-10.1177_14034948221089056 – Supplemental material for Associations between children’s diagnosed mental disorders and educational achievements in Sweden Click here for additional data file. Supplemental material, sj-docx-1-sjp-10.1177_14034948221089056 for Associations between children’s diagnosed mental disorders and educational achievements in Sweden by Cristian Bortes, Karina Nilsson and Mattias Strandh in Scandinavian Journal of Public Health
