# Supplementary material for: Associations between children’s diagnosed mental disorders and educational achievements in Sweden
Source: Scand J Public Health. 2022 Apr 13;50(8):1140–7. doi: 10.1177/14034948221089056 (PMC9720461; doi:10.1177/14034948221089056)
Supplement: sj-docx-1-sjp-10.1177_14034948221089056 – Supplemental material for Associations between children’s diagnosed mental disorders and educational achievements in Sweden [file sj-docx-1-sjp-10.1177_14034948221089056.docx]

**Appendix**

eTable 1a. Frequencies showing which diagnoses occur together among males

eTable 1b. Frequencies showing which diagnoses occur together among females

eTable 2. Associations between mental disorders and overall grade sum

eTable 3. Associations between mental disorders and ineligibility for upper secondary education

**eTable 1a. Frequencies showing which diagnoses occur together among males**

| Mood disorder | Unipolar depression | Anxiety disorder | OCD | Eating disorder | ADHD | Freq. | Percent |
| --- | --- | --- | --- | --- | --- | --- | --- |
| 0 | 0 | 0 | 0 | 0 | 0 | 117 776 | 86.26 |
| 0 | 0 | 0 | 0 | 0 | 1 | 13 983 | 10.24 |
| 0 | 0 | 1 | 0 | 0 | 0 | 1313 | 0.96 |
| 0 | 1 | 0 | 0 | 0 | 0 | 833 | 0.61 |
| 0 | 0 | 1 | 0 | 0 | 1 | 716 | 0.52 |
| 0 | 1 | 0 | 0 | 0 | 1 | 466 | 0.34 |
| 0 | 0 | 0 | 1 | 0 | 0 | 290 | 0.21 |
| 0 | 1 | 1 | 0 | 0 | 0 | 286 | 0.21 |
| 0 | 0 | 0 | 1 | 0 | 1 | 175 | 0.13 |
| 0 | 0 | 0 | 0 | 1 | 0 | 146 | 0.11 |
| 0 | 1 | 1 | 0 | 0 | 1 | 146 | 0.11 |
| 0 | 0 | 1 | 1 | 0 | 0 | 63 | 0.05 |
| 0 | 0 | 1 | 1 | 0 | 1 | 55 | 0.04 |
| 1 | 0 | 0 | 0 | 0 | 1 | 48 | 0.04 |
| 0 | 0 | 0 | 0 | 1 | 1 | 38 | 0.03 |
| 1 | 0 | 0 | 0 | 0 | 0 | 35 | 0.03 |
| 0 | 1 | 0 | 1 | 0 | 0 | 18 | 0.01 |
| 1 | 0 | 1 | 0 | 0 | 1 | 16 | 0.01 |
| 1 | 1 | 0 | 0 | 0 | 0 | 14 | 0.01 |
| 1 | 1 | 0 | 0 | 0 | 1 | 13 | 0.01 |
| 0 | 1 | 0 | 0 | 1 | 0 | 12 | 0.01 |
| 0 | 0 | 1 | 0 | 1 | 0 | 10 | 0.01 |
| 1 | 1 | 1 | 0 | 0 | 0 | 10 | 0.01 |
| 0 | 0 | 0 | 1 | 1 | 0 | 7 | 0.01 |
| 0 | 1 | 1 | 1 | 0 | 1 | 7 | 0.01 |
| 0 | 1 | 1 | 0 | 1 | 0 | 6 | 0.00 |
| 1 | 0 | 1 | 0 | 0 | 0 | 5 | 0.00 |
| 0 | 0 | 1 | 0 | 1 | 1 | 4 | 0.00 |
| 0 | 1 | 0 | 0 | 1 | 1 | 4 | 0.00 |
| 0 | 0 | 0 | 1 | 1 | 1 | 2 | 0.00 |
| 0 | 1 | 1 | 1 | 1 | 0 | 2 | 0.00 |
| 1 | 0 | 1 | 1 | 0 | 1 | 2 | 0.00 |
| 0 | 0 | 1 | 1 | 1 | 0 | 1 | 0.00 |
| 1 | 0 | 0 | 1 | 0 | 1 | 1 | 0.00 |
| 1 | 0 | 1 | 0 | 1 | 0 | 1 | 0.00 |
| 1 | 0 | 1 | 1 | 0 | 0 | 1 | 0.00 |
| 1 | 1 | 1 | 1 | 1 | 1 | 1 | 0.00 |

**eTable 1b. Frequencies showing which diagnoses occur together among females**

| Mood disorder | Unipolar depression | Anxiety disorder | OCD | Eating disorder | ADHD | Freq. | Percent |
| --- | --- | --- | --- | --- | --- | --- | --- |
| 0 | 0 | 0 | 0 | 0 | 0 | 112 829 | 86.71 |
| 0 | 0 | 0 | 0 | 0 | 1 | 5921 | 4.55 |
| 0 | 0 | 1 | 0 | 0 | 0 | 3228 | 2.48 |
| 0 | 1 | 0 | 0 | 0 | 0 | 1902 | 1.46 |
| 0 | 0 | 0 | 0 | 1 | 0 | 1541 | 1.18 |
| 0 | 1 | 1 | 0 | 0 | 0 | 1090 | 0.84 |
| 0 | 0 | 1 | 0 | 0 | 1 | 924 | 0.71 |
| 0 | 1 | 0 | 0 | 0 | 1 | 530 | 0.41 |
| 0 | 1 | 1 | 0 | 0 | 1 | 383 | 0.29 |
| 0 | 0 | 0 | 1 | 0 | 0 | 306 | 0.24 |
| 0 | 0 | 1 | 0 | 1 | 0 | 204 | 0.16 |
| 0 | 1 | 1 | 0 | 1 | 0 | 188 | 0.14 |
| 0 | 0 | 0 | 0 | 1 | 1 | 122 | 0.09 |
| 0 | 0 | 1 | 1 | 0 | 0 | 116 | 0.09 |
| 0 | 1 | 1 | 0 | 1 | 0 | 112 | 0.09 |
| 0 | 0 | 0 | 1 | 0 | 1 | 86 | 0.07 |
| 1 | 0 | 0 | 0 | 0 | 0 | 67 | 0.05 |
| 0 | 0 | 1 | 1 | 0 | 1 | 60 | 0.05 |
| 1 | 0 | 0 | 0 | 0 | 1 | 42 | 0.03 |
| 1 | 0 | 1 | 0 | 0 | 0 | 42 | 0.03 |
| 0 | 0 | 0 | 1 | 1 | 0 | 41 | 0.03 |
| 1 | 1 | 0 | 0 | 0 | 0 | 39 | 0.03 |
| 1 | 1 | 1 | 0 | 0 | 0 | 38 | 0.03 |
| 0 | 1 | 1 | 0 | 1 | 1 | 36 | 0.03 |
| 0 | 0 | 1 | 0 | 1 | 1 | 33 | 0.03 |
| 0 | 1 | 0 | 0 | 1 | 1 | 30 | 0.02 |
| 0 | 1 | 1 | 1 | 0 | 1 | 27 | 0.02 |
| 1 | 0 | 1 | 0 | 0 | 1 | 24 | 0.02 |
| 0 | 1 | 1 | 1 | 0 | 0 | 23 | 0.02 |
| 0 | 1 | 1 | 1 | 0 | 1 | 19 | 0.01 |
| 1 | 1 | 1 | 0 | 0 | 1 | 16 | 0.01 |
| 0 | 0 | 1 | 1 | 1 | 0 | 15 | 0.01 |
| 1 | 1 | 0 | 0 | 0 | 1 | 12 | 0.01 |
| 0 | 1 | 0 | 1 | 0 | 1 | 9 | 0.01 |
| 0 | 0 | 1 | 1 | 1 | 1 | 8 | 0.01 |
| 1 | 1 | 1 | 0 | 1 | 0 | 8 | 0.01 |
| 0 | 1 | 0 | 1 | 1 | 0 | 7 | 0.01 |
| 0 | 1 | 1 | 1 | 1 | 0 | 5 | 0.00 |
| 1 | 0 | 1 | 0 | 1 | 1 | 5 | 0.00 |
| 0 | 0 | 0 | 1 | 1 | 1 | 4 | 0.00 |
| 0 | 1 | 0 | 1 | 1 | 1 | 4 | 0.00 |
| 1 | 0 | 0 | 0 | 1 | 0 | 4 | 0.00 |
| 1 | 0 | 1 | 1 | 0 | 0 | 4 | 0.00 |
| 1 | 1 | 1 | 1 | 0 | 0 | 4 | 0.00 |
| 1 | 1 | 0 | 0 | 1 | 0 | 3 | 0.00 |
| 0 | 1 | 1 | 1 | 1 | 1 | 2 | 0.00 |
| 1 | 0 | 0 | 1 | 0 | 0 | 2 | 0.00 |
| 1 | 0 | 1 | 1 | 0 | 1 | 2 | 0.00 |
| 1 | 1 | 0 | 0 | 1 | 1 | 2 | 0.00 |
| 1 | 1 | 1 | 1 | 0 | 1 | 2 | 0.00 |
| 1 | 0 | 0 | 1 | 0 | 1 | 1 | 0.00 |
| 1 | 0 | 1 | 1 | 1 | 1 | 1 | 0.00 |
| 1 | 1 | 0 | 1 | 0 | 1 | 1 | 0.00 |
| 1 | 1 | 1 | 0 | 1 | 1 | 1 | 0.00 |

**eTable 2. Associations between mental disorders and overall grade sum**

|  | Standardised mean grade difference (95% CI) | | | |
| --- | --- | --- | --- | --- |
|  | Model 1^a^ | | Model 2^b^ | |
|  | Male | Female | Male | Female |
|  |  |  |  |  |
| Mood disorder ^c^ | -0.95 (-1.08 to -0.81) | -1.03 (-1.13 to -0.94) | -0.93 (-1.06 to -0.81) | -0.99 (-1.08 to -0.90) |
| *r*^2^ | 0.0013 | 0.0033 | 0.1667 | 0.1596 |
|  |  |  |  |  |
| Unipolar depression ^d^ | -0.89 (-0.93 to -0.85) | -0.80 (-0.83 to -0.78) | -0.88 (-0.92 to -0.85) | -0.77 (-0.79 to -0.75) |
| *r*^2^ | 0.0137 | 0.0271 | 0.1789 | 0.1814 |
|  |  |  |  |  |
| Anxiety disorder ^e^ | -0.77 (-0.80 to -0.73) | -0.83 (-0.85 to -0.80) | -0.73 (-0.76 to -0.70) | -0.78 (-0.80 to -0.76) |
| *r*^2^ | 0.0145 | 0.0401 | 0.1785 | 0.1919 |
|  |  |  |  |  |
| OCD ^f^ | -0.27 (-0.34 to -0.20) | -0.36 (-0.43 to -0.30) | -0.33 (-0.39 to -0.27) | -0.42 (-0.48 to -0.37) |
| *r*^2^ | 0.0004 | 0.0009 | 0.1661 | 0.1578 |
|  |  |  |  |  |
| Eating disorder ^g^ | -0.12 (-012 to -0.00) | 0.13 (0.09 to 0.16) | -0.15 (-0.25 to -0.04) | -0.02 (-0.01 to 0.05) |
| *r*^2^ | 0.0000 | 0.0004 | 0.1655 | 0.1565 |
|  |  |  |  |  |
| ADHD ^h^ | -0.71 (-0.73 to -0.70) | -0.97 (-0.99 to -0.95) | -0.62 (-0.64 to -0.61) | -0.86 (-0.88 to -0.85) |
| *r*^2^ | 0.0664 | 0.0702 | 0.2158 | 0.2121 |

^a^ Crude model

^b^ Adjusted for family disposable income and parental educational level

ICD-10 codes: ^c^ F30-F39, ^d^ F32-F33, ^e^ F40-F48, ^f^ F42, ^g^ F50, ^h^ F90

**eTable 3. Associations between mental disorders and ineligibility for upper secondary education**

|  | ORs for ineligibility (95% CI) | | | |
| --- | --- | --- | --- | --- |
|  | Model 1^a^ | | Model 2^b^ | |
|  | Male | Female | Male | Female |
|  |  |  |  |  |
| Mood disorder ^c^ | 4.93 (3.55 to 6.86) | 5.43 (4.29 to 6.88) | 5.81 (4.09 to 8.26) | 6.05 (4.70 to 7.79) |
| *r*^2^ | 0.0008 | 0.0021 | 0.0989 | 0.1013 |
|  |  |  |  |  |
| Unipolar depression ^d^ | 4.32 (3.90 to 4.77) | 3.81 (3.54 to 4.09) | 5.06 (4.54 to 5.63) | 4.09 (3.78 to 4.41) |
| *r*^2^ | 0.0076 | 0.0146 | 0.1063 | 0.1138 |
|  |  |  |  |  |
| Anxiety disorder ^e^ | 4.02 (3.69 to 4.38) | 4.25 (4.00 to 4.52) | 4.35 (3.97 to 4.78) | 4.40 (4.12 to 4.69) |
| *r*^2^ | 0.0095 | 0.0242 | 0.1076 | 0.1223 |
|  |  |  |  |  |
| OCD ^f^ | 2.65 (2.14 to 3.27) | 2.32 (1.92 to 2.82) | 2.65 (2.14 to 3.27) | 3.14 (2.57 to 3.85) |
| *r*^2^ | 0.0006 | 0.0008 | 0.0988 | 0.1005 |
|  |  |  |  |  |
| Eating disorder ^g^ | 1.96 (1.39 to 2.75) | 0.99 (0.86 to 1.16) | 2.14 (1.49 to 3.08) | 1.39 (1.19 to 1.63) |
| *r*^2^ | 0.0001 | 0.0000 | 0.0982 | 0.0994 |
|  |  |  |  |  |
| ADHD ^h^ | 4.20 (4.03 to 4.39) | 5.84 (5.54 to 6.16) | 3.91 (3.74 to 4.08) | 5.38 (5.09 to 5.69) |
| *r*^2^ | 0.0457 | 0.0486 | 0.1361 | 0.1403 |

^a^ Crude model

^b^ Adjusted for family disposable income and parental educational level

ICD-10 codes: ^c^ F30-F39, ^d^ F32-F33, ^e^ F40-F48, ^f^ F42, ^g^ F50, ^h^ F90
